# Artificial intelligence in pediatric nephrology: current applications and emerging frameworks for evidence generation

**DOI:** 10.1007/s00467-026-07223-8

**Published:** 2026-02-25

**Authors:** Giovanni Pellegrino, Giovanni F. M. Strippoli

**Affiliations:** 1 Department of Precision and Regenerative Medicine and Ionian Area (DIMEPRE-J), University of Bari Aldo Moro, Bari, Italy; 2 Sydney School of Public Health, The University of Sydney, Sydney, NSW Australia

**Keywords:** Artificial intelligence, Machine learning, Pediatric nephrology, Chronic kidney disease, Dialysis, Transplantation, Digital twins

## Abstract

Artificial intelligence (AI) is rapidly transforming many areas of medicine. Pediatric nephrology may be a field of particular relevance due to its data-rich environments, rarity of diseases, long-term follow-up needs, and persistent evidence gaps. Traditional clinical research methods, including randomized controlled trials, are often difficult to conduct in pediatric nephrology because of small sample sizes, ethical constraints, heterogeneity of disease, and long latency to clinically meaningful outcomes. AI-based methods offer complementary approaches to address these challenges, spanning diagnostic support, prognostic modeling, imaging analysis, treatment personalization, and innovative frameworks for evidence generation. This narrative review provides a focused overview of AI applications that are already available or emerging in pediatric nephrology, integrating lessons from broader nephrology research on real-world data and digital-twin technologies. We refer to established and near-term applications in chronic kidney disease, dialysis, transplantation, imaging, pathology, and genomics in children. We then discuss future directions, including AI-enabled virtual trials and digital twins as potential tools to extend evidence to pediatric populations traditionally excluded from trials. Ethical, regulatory, and equity considerations specific to children are highlighted. We conclude that AI should be viewed as an adjunct, rather than a replacement to conventional clinical research, with particular promise for advancing precision, inclusivity, and timeliness of evidence in pediatric nephrology.

**Graphical abstract:**

**Figure:**
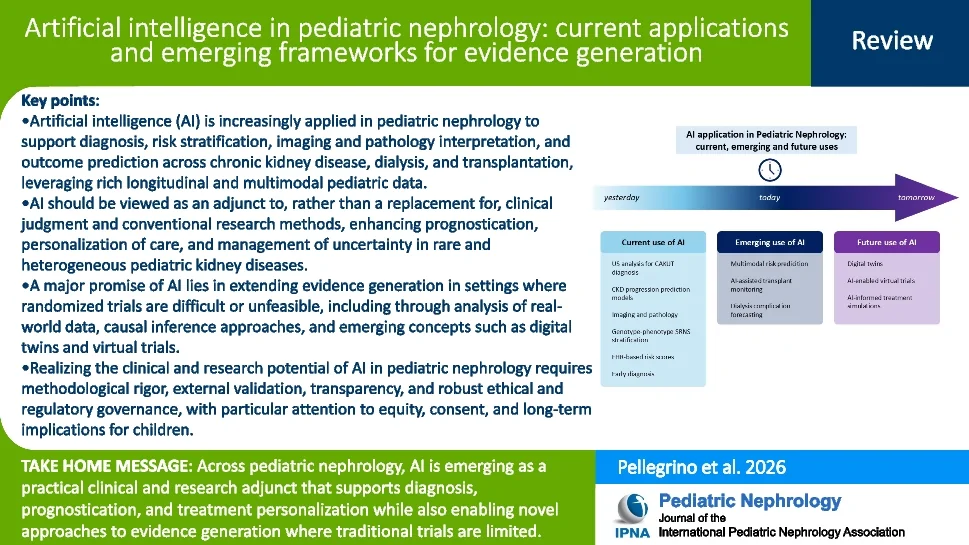
A higher resolution version of the Graphical abstract is available as Supplementary information

**Supplementary Information:**

The online version contains supplementary material available at https://doi.org/10.1007/s00467-026-07223-8.

## Case scenario: the uncertainty of clinical decisions in pediatric nephrology

A 6-year-old child presents with progressive kidney dysfunction following a diagnosis of bilateral congenital anomalies of the kidney and urinary tract identified antenatally. Despite early nephrology follow-up, optimized blood pressure control, and avoidance of nephrotoxins, kidney function continues to decline unpredictably. Genetic testing identifies several variants of uncertain significance, none clearly diagnostic. There is no randomized trial to guide the timing of dialysis access placement, limited pediatric evidence to inform long-term prognostication, and no reliable way to estimate the likelihood of preserving native kidney function through adolescence. Counseling the family relies on extrapolation from heterogeneous cohorts, expert opinion, and the clinician’s experience with similar, but never identical, patients.

This scenario illustrates the profound uncertainty that characterizes many clinical decisions in pediatric nephrology. Children with kidney disease often present with rare, heterogeneous conditions in which disease mechanisms are incompletely understood and outcomes unfold over long time horizons. Conventional approaches to evidence generation struggle in this context. Randomized controlled trials (RCTs), while foundational for causal inference, are frequently unfeasible or underpowered because of small patient populations, ethical constraints on randomization, prolonged follow-up requirements, and substantial logistical and financial barriers.

Long-term follow-up represents an additional challenge in pediatric nephrology, as many clinically meaningful outcomes, such as progression to kidney failure, growth impairment, or cardiovascular complications, may only become apparent over years or decades, limiting the feasibility and timeliness of conventional clinical trials. As a result, clinical management is commonly informed by observational studies, extrapolation from adult populations, or expert consensus, each of which carries important limitations.

In parallel with these challenges, pediatric nephrology has become increasingly data-rich. Large longitudinal cohorts and registries, including the Chronic Kidney Disease in Children (CKiD) study, the North American Pediatric Renal Trials and Collaborative Studies (NAPRTCS), the European Society for Pediatric Nephrology (ESPN)/European Renal Association (ERA) registries, and multiple disease-specific international consortia, have generated extensive clinical, laboratory, imaging, and outcome data [1–5]. However, the full potential of these data remains underexploited in routine clinical care and research.

Artificial intelligence (AI), encompassing machine learning (ML), deep learning, and data-driven causal inference methods, offers new opportunities to complement traditional clinical research paradigms in this setting. Beyond direct clinical applications, AI is also increasingly relevant to pediatric nephrology research and education, including hypothesis generation, trial emulation, biomarker discovery, and the training of clinicians to interpret complex data-driven tools. Rather than relying on static risk factors or single-time-point assessments, AI-based approaches can integrate high-dimensional, longitudinal, and multimodal data to model complex disease trajectories and treatment responses. In nephrology more broadly, AI has been applied to risk prediction, disease classification, imaging interpretation, and outcome forecasting. In adult nephrology, methodological innovations such as target trial emulation and digital twin–based simulations have been proposed as pragmatic solutions to clinical questions that are difficult or unethical to address through conventional RCTs [6, 7]. These approaches may be particularly relevant to pediatrics, where evidence gaps are even more pronounced. The field is undergoing rapid evolution and there already are emerging reporting standards, including TRIPOD-AI, CONSORT-AI, and SPIRIT-AI, to provide guidance on the reporting of AI–based prediction models and interventions [8–10]. These efforts are likely to make results of currently developing studies more transparent and easier to interpret by the users of research results. To support accessibility for readers without a technical background, key AI methods and reporting guidelines referenced in this manuscript are summarized in plain language in Supplementary Table 1.

Revisiting the clinical scenario above, AI-enabled approaches would not eliminate uncertainty or replace clinical judgment but could support more informed decision-making by integrating longitudinal clinical, imaging, and genetic data. By estimating individualized risk trajectories and quantifying uncertainty, such models may enrich discussions with families and support more personalized care, while functioning strictly as adjuncts to existing evidence frameworks.

**Fig. 1:**
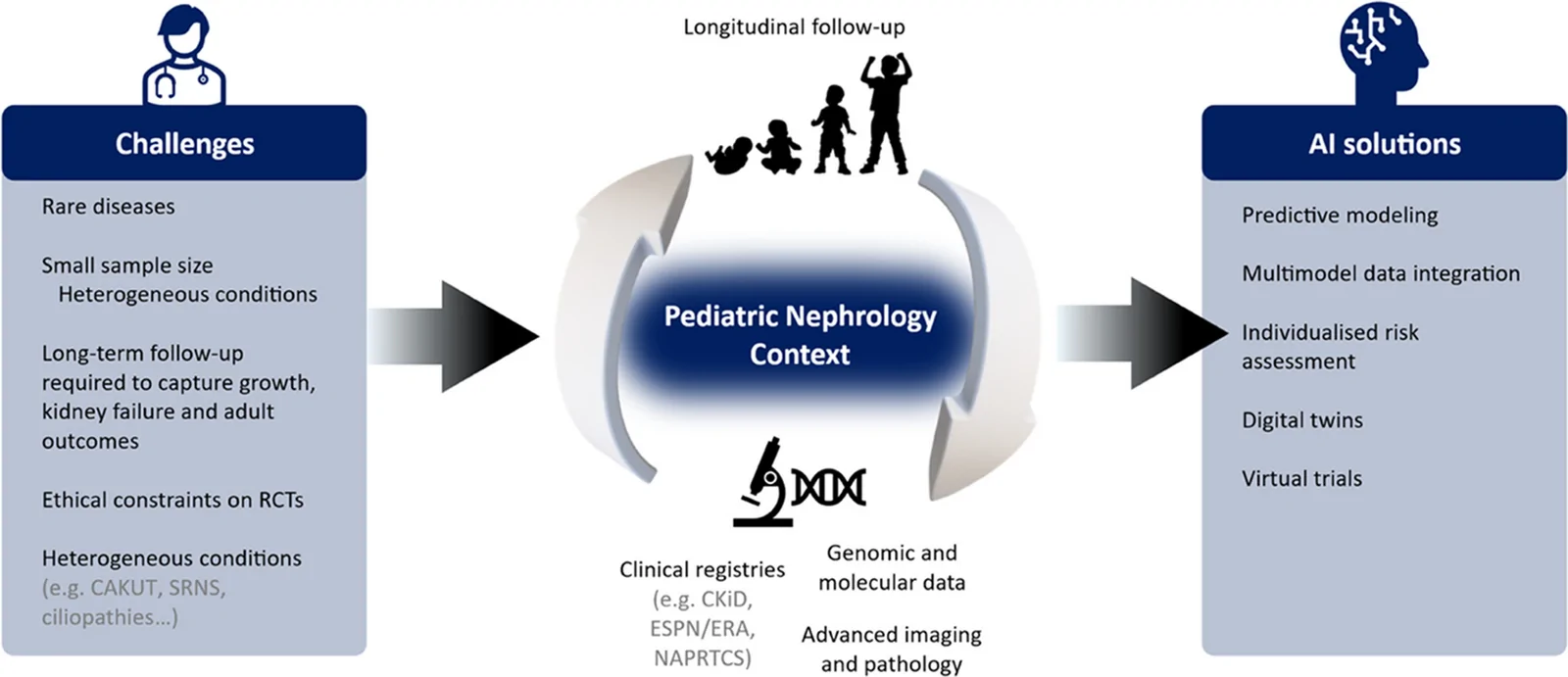
Challenges and potential AI solutions in pediatric nephrology. AI: Artificial Intelligence; CAKUT: Congenital Anomalies of Kidney and Urinary Tract; CKiD: Chronic Kidney Disease in Children; ERA: European Renal Association; ESPN: European Society of Pediatric Nephrology; NAPRTCS: North American Pediatric Renal Trials and Collaborative Studies; RCTs: Randomized Controlled Trials; SRNS: Steroid-Resistant Nephrotic Syndrome

This narrative review complements recent reviews published on pediatric nephrology that primarily catalog AI applications or provide technical primers on machine learning methods [11, 12]. Here, we elaborate on the role of AI in pediatric nephrology. We focus on how AI may address persistent challenges in evidence generation, uncertainty, and decision-making that are specific to pediatric nephrology. Building on methodological insights from adult nephrology and broader AI research, we aim to summarize AI applications that are already available or approaching clinical implementation in pediatric kidney care; highlight emerging and near-term innovations with relevance to children; and discuss ethical, regulatory, and implementation challenges unique to pediatric populations. Through this lens, we explore how AI may help extend evidence generation, support precision medicine, and address long-standing unmet needs in pediatric nephrology while maintaining a cautious, rigorous, and patient-centered approach (Fig. 1).

To support accessibility, a glossary of key AI terms and reporting guidelines referenced throughout the manuscript are summarized in plain language in Supplementary Table 1.

## Methods of AI relevant to pediatric nephrology

AI in medicine encompasses a broad range of computational approaches, including supervised learning, unsupervised learning, reinforcement learning, and hybrid causal-ML frameworks (Table 1) [6, 7, 13–22].

**Table 1:** Main computational approaches used in AI and their meaning in medical practice. Conceptual synthesis informed by methodological literature on medical AI and clinical prediction modeling [6, 7, 13–22]

AI approach	What it means in simple terms	Type of data used	Typical medical question it addresses	Example relevance in pediatric nephrology
Supervised learning	The computer learns by example, using past patient data where the outcome is already known	Clinical data linked to known outcomes	What is likely to happen to this patient	Predicting progression of kidney disease based on previous children with similar characteristics
Unsupervised learning	The computer looks for patterns without being told what the correct answer is	Clinical or biological data without predefined outcomes	Are there hidden subgroups or patterns	Identifying previously unrecognized subtypes of kidney disease
Reinforcement learning	The computer learns by trial and error, receiving feedback on whether decisions lead to better outcomes	Sequential clinical decisions and outcomes	What decision strategy works best over time	Optimizing dialysis treatment adjustments over repeated sessions
Hybrid causal–machine learning frameworks	Combines pattern recognition with methods designed to estimate cause-and-effect relationships	Observational clinical data with defined interventions	What would happen if we chose one treatment instead of another	Comparing outcomes under different treatment strategies when trials are not feasible

To date, most AI applications in pediatric nephrology rely on supervised learning, where models are trained to predict outcomes or classify disease states using labeled data derived from electronic health records (EHRs), registries, imaging studies, pathology specimens, or omics platforms.

A more detailed description of specific machine learning architectures and image-based methods has been addressed elsewhere [11, 12]. These approaches are applied within the specific methodological constraints of pediatric nephrology and may complement existing evidence-generation frameworks. Several intrinsic features of pediatric nephrology make it particularly amenable to AI-based approaches. First, children with kidney disease are often followed from infancy through adolescence, generating long, well-annotated longitudinal datasets. Second, routine care involves repeated laboratory testing and imaging, creating dense time-series data. Third, pediatric kidney diseases are disproportionately influenced by genetic and developmental factors, increasing the relevance of multi-omics integration. Finally, clinical decision-making frequently requires synthesis of heterogeneous data sources to support individualized care, a task well aligned with AI methodologies.

At the same time, important methodological challenges must be addressed. Pediatric datasets are typically smaller than adult cohorts, increasing the risk of overfitting and unstable estimates. Rare disease phenotypes and adverse outcomes lead to pronounced class imbalance, requiring careful model design, appropriate evaluation metrics, and transparent reporting. External validation across centers and health systems is essential yet remains uncommon in pediatric AI literature.

## The present: AI applications already available in pediatric nephrology

**Fig. 2:**
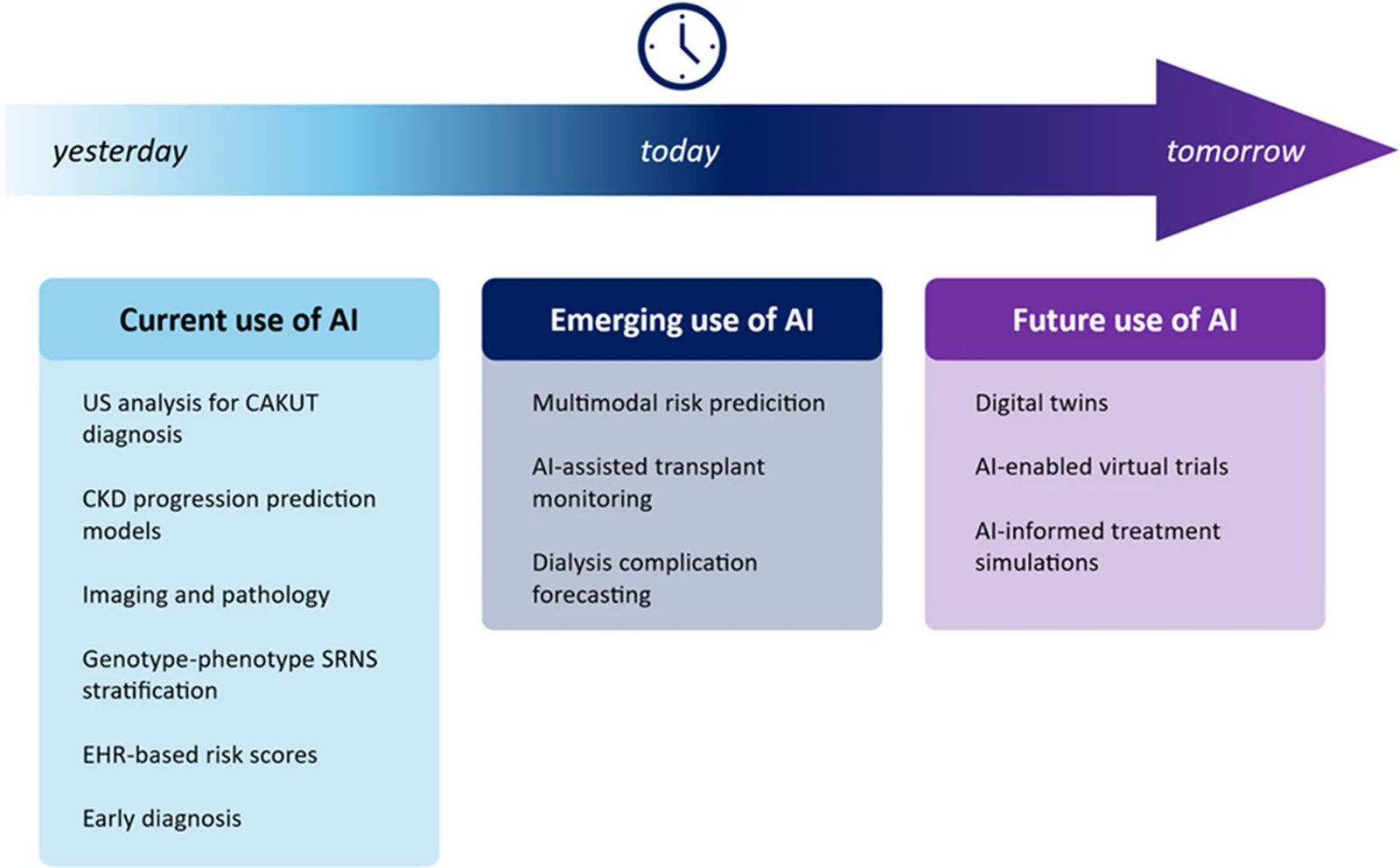
AI applications across the pediatric nephrology care continuum: current and emerging use cases. AI: Artificial intelligence; CAKUT: Congenital Anomalies of Kidney and Urinary Tract; CKD: Chronic Kidney Disease; EHR: Electronic Health Records; SRNS: Steroid-Resistant Nephrotic Syndrome; US: Ultrasound

An overview of current and emerging AI applications across major domains of pediatric nephrology is provided in Fig. 2. These encompass diagnostic imaging and pattern recognition, risk stratification and predictive modeling, dialysis management and genomic and omics integration [13–15, 23–38].

### Chronic kidney disease and progression modeling

Predicting progression of chronic kidney disease (CKD) in children is a central challenge in pediatric nephrology. Traditional risk models have relied on linear regression or Cox proportional hazards approaches using baseline estimated glomerular filtration rate (eGFR), proteinuria, blood pressure, and underlying diagnosis. However, these methods often fail to capture nonlinear interactions and temporal dynamics.

ML models incorporating longitudinal data have demonstrated improved predictive performance for CKD progression in pediatric cohorts [16]. Analyses using data from the CKiD study have shown that ML-based approaches can more accurately predict eGFR decline and progression to kidney failure compared with traditional statistical models, particularly when incorporating time-varying laboratory values, growth parameters, and medication exposure. Similar approaches have been applied to foretell hospitalization risk and cardiovascular complications, which remain major contributors to morbidity in children with CKD.

These models have potential clinical utility in identifying high-risk children earlier in the disease course, informing the timing of referral for transplant evaluation, dialysis access planning, and enrollment in interventional trials. Importantly, explainability techniques such as SHAP (Shapley additive explanations) values are increasingly used to support clinical interpretability and trust.

### Congenital anomalies of the kidney and urinary tract

Congenital anomalies of the kidney and urinary tract (CAKUT) represent the most common cause of CKD in children and encompasses a highly heterogeneous spectrum of structural abnormalities. Prognostication based on prenatal and postnatal imaging remains imprecise. AI-based image analysis has been explored to automate kidney ultrasound interpretation, identify complex morphologic patterns, and predict long-term functional outcomes [13, 14, 17, 18, 23–25, 28, 29].

Studies using ML to analyze ultrasound features have demonstrated improved model performance for prediction of postnatal kidney function and need for surgical intervention compared with conventional radiologic descriptors [26, 27]. Integration of imaging features with clinical variables and genetic data may further refine phenotypic classification and support personalized counseling for families. Although most existing studies are small and single-center, they illustrate the feasibility of AI-assisted risk stratification in CAKUT.

### Pediatric dialysis

Children receiving dialysis represent a small but clinically complex population characterized by high morbidity and frequent complications. Despite limited patient numbers, dialysis generates dense longitudinal data, including biochemical parameters, fluid status, growth metrics, and treatment modifications. AI approaches have been applied to predict intradialytic hypotension, fluid overload, and growth outcomes, drawing on experience from adult dialysis populations [19, 20, 22].

In pediatrics, predictive models could support individualized dialysis prescriptions, early identification of patients at risk for complications, and benchmarking of quality-of-care metrics across centers. Moreover, advanced causal inference methods applied to observational dialysis data may help evaluate alternative treatment strategies, such as modality choice or ultrafiltration targets, in settings where RCTs are unfeasible.

### Kidney transplantation

Pediatric kidney transplantation offers the best long-term outcomes for children with kidney failure, yet graft survival remains limited by rejection, infection, and medication-related toxicity. AI models have been developed to predict graft survival and acute rejection using donor and recipient characteristics, immunologic profiles, and post-transplant laboratory trends [39, 40].

Emerging work integrates EHR-derived adherence measures, pharmacokinetic data, and longitudinal biomarkers to support personalized immunosuppression strategies. Deep learning–based analysis of transplant biopsies has shown promise in detecting subclinical rejection and fibrosis earlier than conventional pathology scoring systems, potentially enabling preemptive intervention.

### Imaging and digital pathology

AI–based imaging and digital pathology techniques in nephrology have already been reviewed in detail [11, 12]. In pediatric nephrology, these approaches may be particularly valuable for addressing specific structural and organizational challenges, including limited access to subspecialty expertise, interobserver variability, and the need for longitudinal assessment across developmental stages.

AI-assisted analysis of kidney biopsies has demonstrated high accuracy in quantifying glomerular and tubular lesions, interstitial fibrosis, and inflammatory infiltrates [21]. Although most validated studies to date have been conducted in adult populations, adaptation to pediatric datasets is underway and may support more standardized histopathologic assessment across centers. In this context, the potential value of AI lies less in diagnostic automation and more in improving consistency, comparability, and scalability of imaging-based assessments over time.

## Practical questions frequently posed by clinicians about AI in current pediatric nephrology practice and examples of publicly available AI tools relevant to pediatric nephrology

As AI becomes increasingly visible in clinical scenarios, pediatric nephrologists are confronted with practical questions regarding how AI can be accessed, interpreted, and trusted in day-to-day practice. At present, most AI applications relevant to pediatric nephrology are not accessed through conversational general-purpose tools, but rather through disease- or task-specific models embedded in web-based calculators, research platforms, imaging software, or clinical decision support systems linked to EHRs. General-purpose large language models may support educational activities or high-level information synthesis, but they are not designed or validated to provide patient-specific predictions, risk estimates, or therapeutic recommendations in pediatric nephrology. Importantly, AI outputs should never be interpreted as definitive clinical judgments. Predictions and risk estimates represent probabilistic assessments based on prior data and assumptions, and their reliability depends on data quality, model validation, and clinical context. Human oversight therefore remains essential. Clinicians are responsible for evaluating whether AI-derived outputs are plausible, consistent with the individual patient’s trajectory, and appropriate for the clinical setting.

AI-based predictions are also dynamic rather than static. When a child’s clinical course deviates from expected trajectories — for example after transplantation, during intercurrent illness, or following changes in therapy — model outputs should be updated by incorporating new follow-up data rather than treated as fixed forecasts. This adaptive use of AI mirrors clinical reasoning and underscores that AI systems are tools to support, not replace, longitudinal clinical judgment.

Table 2 outlines several practical questions and answers for clinicians using AI in pediatric nephrology [6–10]. Supplementary Table 2 lists examples of publicly available AI tools relevant to pediatric nephrology.

**Table 2:** Practical questions and answers for clinicians using AI in pediatric nephrology. Questions and responses reflect common issues raised in the clinical implementation of AI-based decision support systems and are informed by existing reporting standards and clinical governance literature [6–10]

Clinical question	Practical answer	Key caveat
How can clinicians access AI tools today?	Most AI tools are accessed through task-specific systems such as web-based calculators, imaging software, research platforms, or clinical decision support tools integrated into electronic health records	Availability varies widely, and local validation is often required before clinical use
Can general-purpose AI tools (e.g., ChatGPT) be used for clinical decisions?	No. General-purpose large language models may support education or information synthesis but are not designed for patient-specific prediction or treatment guidance	Outputs are not clinically validated, traceable, or accountable
Can AI be queried iteratively with simple questions, like a conversation?	Most clinical AI systems rely on structured data inputs rather than free-text dialogue, although predictions can be updated as new data become available	Conversational interaction should not be confused with clinical decision support
Should clinicians always trust AI outputs?	No. AI provides probabilistic estimates that must be interpreted within clinical context and combined with professional judgment	Blind trust may lead to automation bias and inappropriate decisions
Who is responsible for decisions informed by AI?	Responsibility remains with the clinician and the healthcare system, not the AI tool	Clear accountability is essential for ethical and legal reasons
What if a patient’s follow-up deviates from predicted trajectories?	AI predictions should be updated by incorporating new longitudinal data rather than treated as fixed forecasts	Static models that do not adapt to evolving data have limited clinical value
Is this AI validated in children similar to my patient?	Clinicians should verify whether the model has been validated in pediatric populations comparable in age, diagnosis, and care setting	Limited generalizability may compromise reliability in rare or underrepresented groups
How accurate is the prediction, and how much uncertainty is there?	Clinically useful AI models should report uncertainty estimates or risk ranges, not only point predictions	Apparent precision can be misleading without uncertainty quantification
Does AI add information beyond standard clinical assessment?	AI is most valuable when it provides incremental insight beyond established risk factors and clinical intuition	Models that merely replicate known predictors offer limited benefit
Can AI outputs be explained to patients and families?	Explainability is essential to support shared decision-making, particularly in pediatric care	Black-box predictions are difficult to justify in counseling contexts
What happens if the AI prediction is wrong?	AI outputs should be documented as supportive information rather than determinants of care decisions	AI does not transfer clinical or legal responsibility away from the clinician
Is this AI tool intended for research or routine clinical care?	Many AI tools remain research-only; clinical use requires appropriate regulatory approval and governance	Using research tools in clinical care may carry ethical and legal risks
Will using AI increase clinical workload?	AI adoption should ideally reduce cognitive or operational burden by integrating smoothly into existing workflows	Poorly designed systems may increase workload or alert fatigue
Does AI replace clinical reasoning?	No. AI complements but does not replace clinical expertise, patient preferences, or shared decision-making	AI should inform decisions, not dictate them

## Transitioning to the future: emerging applications and hypotheses on near-term innovations

**Fig. 3:**
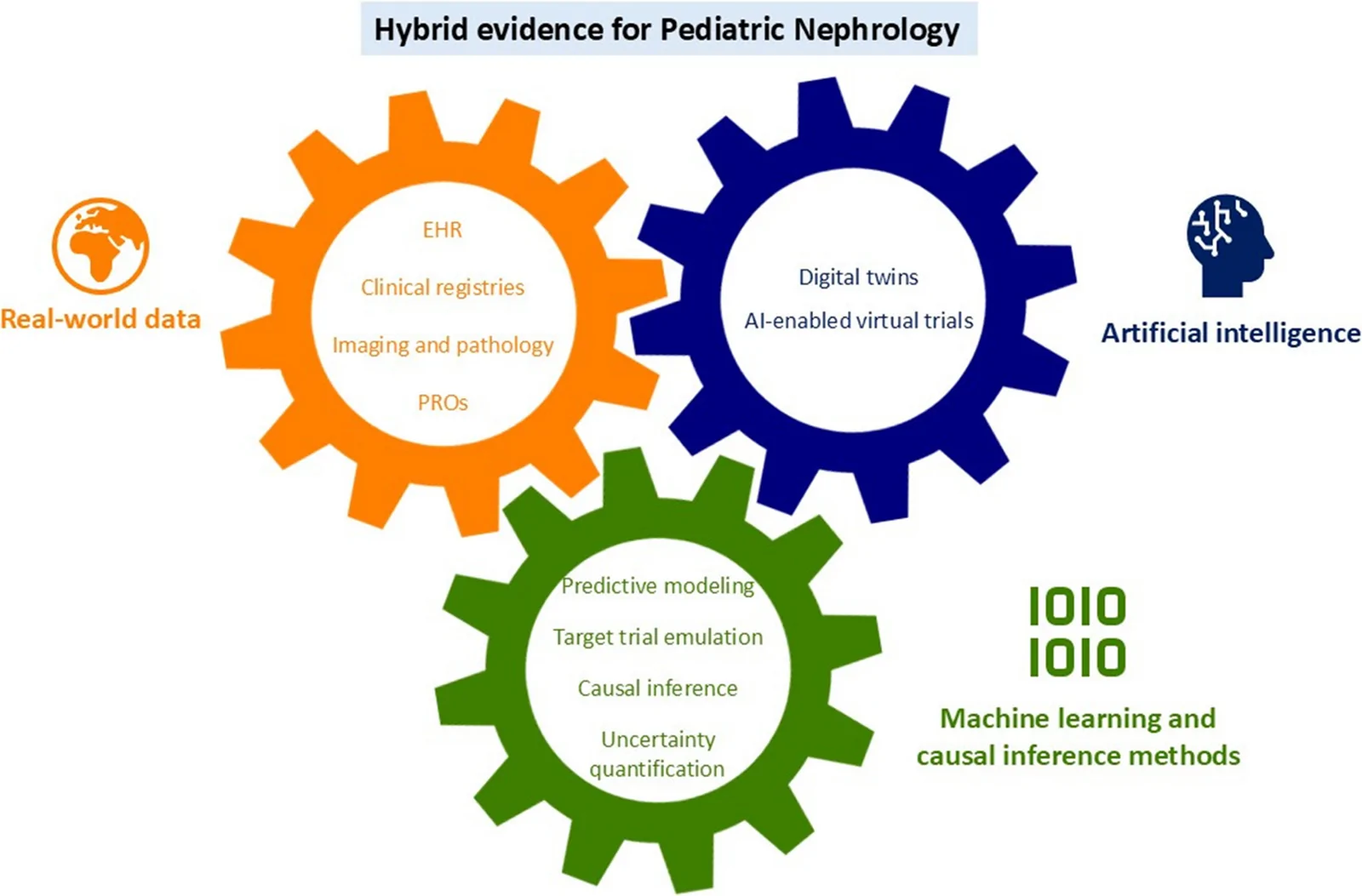
From real-world data to hybrid evidence generation: an AI-enabled ecosystem in pediatric nephrology. AI: Artificial Intelligence; EHR: Electronic Health Records; PRO: Patient-Related Outcome

Table 3 outlines potential future clinical applications of AI in pediatric nephrology. Figure 3 provides a conceptual overview of how real-world data, causal inference, and simulation-based methods may converge to support hybrid evidence generation in pediatric nephrology.

**Table 3:** Potential future clinical applications of AI in pediatric nephrology

Future approach	What it means in clinical terms	Example clinical question	Potential benefit for patients and families	Key requirement for safe use
Patient-specific simulation models	Computer models that mirror an individual child’s disease course	What happens if dialysis is started earlier versus later	More informed counseling	Transparent uncertainty estimates
AI–supported comparative analyses	Comparison of treatment strategies using real-world data	Which dialysis modality leads to better growth	Evidence where trials are not feasible	Careful bias control
Integrated genetic and clinical modeling	Linking genetic findings to outcomes	Is this genetic result clinically meaningful	Precision diagnosis	Multicenter validation
Hybrid evidence frameworks	Combining observational data and simulation	Long-term outcomes beyond trial follow-up	More inclusive evidence	Regulatory oversight

### Integration of genomics and multi-omics data

A substantial proportion of pediatric kidney diseases are monogenic or strongly influenced by genetic variation, making pediatric nephrology one of the clinical specialties most immediately positioned to benefit from AI-enabled multi-omics integration. Next-generation sequencing (NGS) has transformed diagnostic pathways for CAKUT, nephrotic syndrome, ciliopathies, and tubulointerstitial disorders [41]. However, the rapid expansion of genomic testing has outpaced our ability to interpret variants in a clinically actionable manner, particularly in the context of variable penetrance, modifier genes, and developmental influences.

A major downstream consequence of expanded genomic testing in pediatric nephrology is the high prevalence of variants of uncertain significance (VUS), which frequently complicate diagnosis, prognostication, and clinical decision-making in children with rare kidney diseases. This challenge is particularly pronounced in conditions such as CAKUT, steroid-resistant nephrotic syndrome, and ciliopathies, where genotype–phenotype correlations are complex and often incomplete. AI-based approaches offer emerging opportunities to address this gap by integrating variant-level data with deep clinical phenotyping, longitudinal outcomes, and multi-omics signatures. ML models can support probabilistic reclassification of VUS by leveraging large-scale reference datasets, functional predictions, and phenotype–genotype similarity across patients. As these methods mature, AI-assisted variant interpretation may reduce diagnostic uncertainty, support more precise counseling of families, and accelerate the translation of genomic findings into clinically actionable insights in pediatric nephrology, including the progressive reclassification of VUS as pathogenic or likely benign as additional data accumulate. AI and ML approaches are well suited to address this complexity by integrating genomic data with transcriptomic, proteomic, metabolomic, and epigenomic layers, alongside detailed clinical phenotypes. Rather than treating genetic variants as static features, AI models can incorporate dynamic molecular signatures and longitudinal clinical data, enabling a more nuanced understanding of disease mechanisms and trajectories. In pediatric kidney disease, where genotype–phenotype relationships are often nonlinear and context-dependent, such approaches may outperform traditional rule-based interpretation frameworks.

In steroid-resistant nephrotic syndrome (SRNS), for example, ML-based analyses have been used to identify genotype–phenotype correlations and predict response to immunosuppressive therapy, distinguishing children with monogenic disease, who are unlikely to benefit from intensified immunosuppression, from those with immune-mediated forms [41]. Building on this foundation, future multi-omics models could integrate podocyte transcriptomic signatures, circulating proteomic biomarkers, and longitudinal treatment response data to further refine disease subtypes and inform therapeutic decision-making.

Beyond nephrotic syndrome, AI-enabled multi-omics integration holds promise for redefining disease classification across pediatric nephrology. Current diagnostic categories often group biologically heterogeneous conditions under a single clinical label. By identifying molecularly defined subgroups within entities such as CAKUT or pediatric CKD of unknown etiology, AI-driven approaches could support a shift from phenotype-based to mechanism-based classification systems. Such reclassification has direct implications for prognosis, clinical trial design, and regulatory approval of targeted therapies.

From a translational perspective, AI-based multi-omics analysis may also facilitate discovery of novel therapeutic targets and biomarkers. Network-based models can identify key regulatory pathways and molecular interactions that drive disease progression, highlighting candidate targets for drug development or repurposing. In parallel, predictive models trained on integrated omics and clinical data could enable stratification of patients for precision therapies, reducing exposure to ineffective or toxic treatments and improving trial efficiency in small pediatric populations.

Realistically, the near-term clinical impact of multi-omics AI in pediatric nephrology is most likely to emerge in specialized centers and research networks, supported by standardized phenotyping, harmonized data pipelines, and close collaboration between clinicians, geneticists, and data scientists. As costs decline and analytical frameworks mature, selected applications — such as AI-assisted variant interpretation or molecular risk stratification — may become increasingly embedded in routine care.

### Digital twins and virtual trials

Digital twins are computational representations of individual patients that simulate disease trajectories and responses to interventions. They represent a forward-looking approach to evidence generation and clinical decision support. Conceptually, a digital twin integrates clinical history, laboratory trends, imaging, genomics, and treatment data into a dynamic model that evolves over time, allowing simulation of alternative clinical scenarios. Although digital twins remain largely experimental in nephrology, foundational components already exist in the form of longitudinal prediction models, causal inference frameworks, and mechanistic simulations.

In pediatric nephrology, the potential value of digital twins is particularly compelling. Many pediatric kidney diseases are rare, progress slowly, and involve complex interactions between development, treatment exposure, and long-term outcomes. Digital twins could enable computational exploration of “what-if” scenarios that are difficult or impossible to test empirically, such as alternative immunosuppression strategies, timing of dialysis initiation, or long-term consequences of early-life interventions [42, 43]. For individual patients, such simulations could support shared decision-making by illustrating plausible future trajectories under different management strategies.

At the population level, digital twins could underpin virtual or hybrid trials, where simulated cohorts complement real-world observational data and limited RCT evidence. For example, digital twin–based simulations could be used to compare dialysis modalities, transplant allocation strategies, or follow-up protocols in rare pediatric conditions, helping to prioritize hypotheses and optimize trial design. In this context, digital twins are not intended to replace clinical trials, but to extend their reach and inform decisions where traditional trials are unfeasible.

Critically, the credibility of digital twin approaches depends on rigorous development and validation. Models must be trained on high-quality, representative datasets and continuously benchmarked against real-world outcomes and, where available, trial data. Transparent reporting, uncertainty quantification, and explicit acknowledgment of model assumptions are essential to avoid overinterpretation. In pediatric populations, additional safeguards are required to ensure ethical use, protect patient privacy, and prevent amplification of existing biases.

In the foreseeable future, the most realistic applications of digital twins in pediatric nephrology are likely to focus on disease trajectory modeling and comparative effectiveness analyses within well-defined clinical contexts. As methodological standards evolve and computational infrastructure improves, digital twins may become a valuable component of hybrid evidence ecosystems, complementing observational studies, target trial emulation, and conventional clinical trials.

## Ethical, regulatory, equity and access considerations

The application of AI in pediatric nephrology raises a set of interrelated ethical, regulatory, and equity challenges that extend beyond algorithmic performance alone. Children represent a particularly vulnerable population, making issues of informed consent and assent, data ownership, privacy protection, and long-term data stewardship especially critical. In addition, AI models trained on historical or geographically restricted datasets risk perpetuating existing biases if underrepresented populations and low-resource settings are insufficiently captured during model development.

Ethical evaluation of AI in pediatrics must also explicitly address access, cost, and real-world feasibility. Although many AI tools are developed within academic environments and may be technically available at low or no licensing cost, their clinical implementation requires digital infrastructure, standardized data acquisition, local validation, regulatory approval, and ongoing maintenance. As a result, access to these systems is not automatically universal, and the presence of an internet connection alone is insufficient to guarantee meaningful or safe use, particularly in low- and middle-income countries.

Capacity building and education are integral components of ethical AI deployment. AI-based decision support systems cannot be assumed to be self-explanatory, nor should clinicians be expected to adopt them without structured training. Investment in clinician education, interdisciplinary collaboration, and user-centered design is essential to ensure correct interpretation, appropriate clinical integration, and sustained trust. Without such efforts, AI risks widening existing disparities rather than reducing them.

Finally, regulatory frameworks for AI-based clinical decision support in pediatrics remain underdeveloped. Transparency, interpretability, continuous post-deployment monitoring, and clear accountability mechanisms are necessary to safeguard patient safety and equity. Active engagement of patients, families, clinicians, ethicists, and regulators should be considered a prerequisite for responsible development and implementation of AI systems in pediatric nephrology [8–10].

## Future directions and research priorities

Advancing the responsible and effective use of AI in pediatric nephrology will require coordinated progress across data infrastructure, methodological rigor, clinical integration, and governance. Several priority areas can be identified that are both ambitious and realistically achievable within current scientific and regulatory frameworks.

### High-quality, multicenter data infrastructure

A foundational requirement is the development of robust, multicenter data infrastructures that reflect the diversity and longitudinal nature of pediatric kidney disease. Existing registries such as CKiD, NAPRTCS, and international transplant and dialysis databases provide valuable starting points [1–4], but future efforts should emphasize harmonization of data elements, standardized phenotyping, and interoperability across health systems and countries. Integration of routinely collected EHR data with imaging, pathology, and genomic information will be essential to support multi-modal AI models and digital twin approaches.

Concrete examples include the creation of federated data networks that allow model training and validation across institutions without centralizing sensitive pediatric data, and the expansion of disease-specific consortia for rare conditions such as monogenic nephrotic syndrome or pediatric tubulointerstitial disease. Such infrastructures would enable adequately powered analyses, facilitate external validation, and reduce center-specific bias.

### Methodological rigor, validation, and reporting standards

Future AI research in pediatric nephrology must prioritize methodological rigor and transparency. Models should be developed using prespecified protocols, appropriate handling of missing data and class imbalance, and evaluation metrics aligned with clinical relevance rather than purely statistical performance. External validation across geographically and demographically distinct cohorts should be considered a minimum standard before clinical deployment.

Thorough adoption of reporting frameworks will improve reproducibility and interpretability [8–10]. Importantly, explainability methods should be routinely incorporated, particularly for models intended to inform clinical decisions. For example, CKD progression models should clearly communicate which longitudinal features — such as blood pressure trajectories, proteinuria burden, or growth patterns — drive risk estimates, enabling clinicians to contextualize predictions within existing clinical knowledge.

### Integration of patient-reported outcomes and family perspectives

Children with kidney disease and their families experience a substantial burden that is not fully captured by traditional biomedical endpoints. Incorporation of patient-reported outcomes (PROs), such as health-related quality of life, fatigue, school participation, and treatment burden, represents a critical research priority. AI methods are well suited to integrate PROs with clinical and biological data, allowing prediction of outcomes that matter most to patients and families [44].

Future models could, for example, predict not only graft survival after transplantation but also trajectories of quality of life or treatment adherence under different immunosuppression strategies. Actively involving patients and caregivers in defining relevant outcomes and acceptable uses of AI will be essential to ensure that technological advances align with real-world needs and values.

### Hybrid evidence frameworks and causal AI

Given the persistent feasibility challenges of RCTs in pediatric nephrology, future research should embrace hybrid evidence frameworks that combine complementary methodologies. Observational studies and registries can provide real-world data at scale; target trial emulation can strengthen causal inference for specific clinical questions; and AI-based simulations or digital twins can extend insights to rare diseases and long-term outcomes.

Concrete applications include emulating trials to compare dialysis initiation strategies or transplant timing, followed by digital twin–based simulations to explore longer-term consequences beyond observed follow-up. Such approaches should be explicitly designed to complement, rather than compete with conventional trials, and to generate hypotheses that can be tested prospectively where feasible.

### Interdisciplinary collaboration and governance

Sustained collaboration between pediatric nephrologists, data scientists, geneticists, ethicists, patients, and regulators will be essential to translate AI innovations into clinical practice. Early engagement with regulatory bodies can help clarify evidentiary requirements for AI-based decision support tools in pediatric populations, while ethical oversight can ensure appropriate consent processes, data stewardship, and bias mitigation.

Training initiatives that enhance AI literacy among clinicians, alongside structured educational efforts for trainees and researchers, will further support responsible implementation. Ultimately, success will depend not only on technical performance, but on trust, transparency, and demonstrated clinical benefit.

## Conclusions

AI is beginning to influence multiple domains of pediatric nephrology, including risk stratification, imaging and pathology analysis, and outcome prediction after dialysis and transplantation. These applications demonstrate the ability of AI to extract clinically meaningful patterns from complex, longitudinal, and multimodal pediatric data, complementing traditional clinical reasoning and research approaches rather than replacing them.

The greatest potential of AI in pediatric nephrology lies in extending evidence generation to rare diseases, heterogeneous phenotypes, and long-term outcomes that are difficult to study using conventional trials alone. When integrated with high-quality observational data, rigorous causal inference methods, and appropriate governance frameworks, AI-based approaches may support more inclusive, timely, and patient-centered evidence. Realizing this potential will require methodological rigor, transparency, pediatric-specific validation, and sustained collaboration among clinicians, researchers, patients, and regulators.

## Supplementary Information

Below is the link to the electronic supplementary material.

Supplementary file1 (DOCX 20.7 KB)

Graphical abstract (PPTX 166 KB)
