# Supplementary material for: Artificial intelligence in pediatric nephrology: current applications and emerging frameworks for evidence generation
Source: Pediatr Nephrol. 2026 Feb 25;41(11):3691–703. doi: 10.1007/s00467-026-07223-8 (PMC13612611; doi:10.1007/s00467-026-07223-8)
Supplement: Supplementary file 1 [file 467_2026_7223_MOESM1_ESM.docx]

**Supplementary table 1.** Glossary* of key artificial intelligence terms and reporting guidelines.

| **Term** | **Plain-language explanation** |
| --- | --- |
| **Artificial intelligence (AI)** | A broad term describing computer-based systems designed to perform tasks that typically require human intelligence, such as pattern recognition, prediction, or decision support. |
| **Machine learning (ML)** | A subset of AI in which algorithms learn patterns from data to make predictions or classifications without being explicitly programmed for each task. |
| **Deep learning** | A subset of machine learning that uses multi-layer neural networks to learn complex patterns from large datasets, particularly effective for image, signal, and longitudinal data. |
| **Neural networks** | Computational models composed of interconnected layers that learn relationships between inputs and outputs through training. |
| **Convolutional neural networks (CNNs)** | A class of deep learning models designed for image data, capable of identifying relevant visual features such as shapes, textures, and spatial patterns. |
| **Supervised learning** | A machine learning approach in which models are trained using data where the correct outcome is already known, allowing the algorithm to learn how input features relate to outcomes. |
| **Unsupervised learning** | A machine learning approach in which algorithms identify patterns or groupings in data without predefined outcome labels. |
| **Reinforcement learning** | A machine learning approach in which an algorithm learns by trial and error, receiving feedback on actions that lead to better or worse outcomes over time. |
| **Transfer learning** | A technique in which a model developed for one task is adapted for a related task, often allowing effective learning from smaller datasets. |
| **High-dimensional data** | Data that include a large number of variables or features, which can be difficult to analyze using traditional statistical methods but are well suited to AI approaches. |
| **Multimodal data** | Data derived from multiple sources or types, such as clinical records, imaging, laboratory values, and genomic information, analyzed together by AI models. |
| **Longitudinal modeling** | AI methods that incorporate repeated measurements over time to capture disease trajectories and changes in risk or outcomes. |
| **Class imbalance** | A situation in which one outcome or category is much rarer than others in a dataset, which can bias model training and evaluation if not properly addressed. |
| **Overfitting** | A modeling error in which a model performs well on training data but poorly on new data because it has learned noise rather than generalizable patterns. |
| **External validation** | Evaluation of a model using data from a different population, center, or time period than the one used for development, to assess generalizability. |
| **Explainability (interpretability)** | The ability to understand how and why an AI model produces a given prediction, supporting clinical trust and appropriate use. |
| **Shapley additive explanations (SHAP)** | An explainability method that quantifies the contribution of each input variable to an individual prediction, helping clinicians understand why a model produced a given result. |
| **Probabilistic prediction** | An AI output expressed as a likelihood or risk estimate rather than a definite outcome, reflecting uncertainty in predictions. |
| **Uncertainty quantification** | Methods used to estimate and communicate how confident an AI model is in its predictions, rather than providing single point estimates alone. |
| **Automation bias** | The tendency to over-rely on automated system outputs, potentially leading users to discount their own clinical judgment or contradictory evidence. |
| **Target trial emulation** | A causal inference approach that applies principles of randomized trial design to observational data to estimate treatment effects when trials are not feasible. |
| **Digital twins** | Computational models that simulate an individual patient’s disease trajectory over time by integrating clinical, biological, and treatment data. |
| **Federated data networks** | A data architecture that allows AI models to be trained across multiple institutions without sharing raw patient-level data, helping to preserve privacy. |
| **TRIPOD-AI** | A reporting guideline providing recommendations for transparent reporting of studies developing or validating AI-based prediction models. |
| **CONSORT-AI** | An extension of the CONSORT guideline for reporting randomized clinical trials evaluating artificial intelligence–based interventions. |
| **SPIRIT-AI** | An extension of the SPIRIT guideline providing recommendations for reporting protocols of clinical trials involving artificial intelligence interventions. |

*This glossary is intended to support accessibility by defining key artificial intelligence terms and reporting guidelines referenced in the manuscript and is not meant to provide a comprehensive technical overview of artificial intelligence methods.

**Supplementary table 2**. List of publicly available AI-based online tools relevant to pediatric nephrology.

| **Tool name** | **Clinical / research domain** | **Main AI approach** | **Main purpose** | **Accessibility** | **Notes / limitations** |
| --- | --- | --- | --- | --- | --- |
| [**Phen2Gene**](https://phen2gene.wglab.org/)  <https://phen2gene.wglab.org/> | Rare kidney diseases, genomics | ML–based phenotype–gene prioritization | Ranks candidate genes based on Human Phenotype Ontology (HPO) terms | Free, web-based | Useful for gene prioritization; does not provide definitive variant classification |
| **[Exomiser](https://www.sanger.ac.uk/tool/exomiser/)**  <https://www.sanger.ac.uk/tool/exomiser> | Genomic diagnostics | Phenotype-driven variant prioritization using ML scoring | Identifies likely disease-causing variants from exome/genome data | Free (web & local installation) | Performance depends on quality of phenotype annotation |
| **[DeepVariant](https://github.com/google/deepvariant)**  <https://github.com/google/deepvariant> | Genomics | Deep learning (CNNs) | Variant calling from next-generation sequencing data | Free, open source | Requires bioinformatics infrastructure; not disease-specific |
| **[SpliceAI](https://github.com/Illumina/SpliceAI)**  https://github.com/illumina/spliceai | Genomic variant interpretation | Deep neural networks | Predicts splicing effects of genetic variants | Free, web-based & API | Supports VUS interpretation; predictions require expert validation |
| **[AlphaMissense](https://alphamissense.hegelab.org/)**  <https://alphamissense.hegelab.org/> | Protein function / genomics | Deep learning | Predicts pathogenicity of missense variants | Free, web-based | Provides probabilistic scores; not a replacement for clinical interpretation |
| **[Kidney Precision Medicine Project (KPMP) Atlas](https://atlas.kpmp.org/)**  <https://atlas.kpmp.org/> | Kidney tissue biology | AI-assisted multiomics integration | Integrates transcriptomic, proteomic, and imaging data | Free, web-based | Primarily research-focused; limited direct clinical application |
| [**CellProfiler**](https://cellprofiler.org/)  <https://cellprofiler.org> | Renal histopathology | ML-based image analysis | Quantitative analysis of microscopy images | Free, open source | Requires user-defined pipelines and validation |
| [**Ilastik**](https://www.ilastik.org/)  <https://www.ilastik.org> | Renal imaging / histology | Interactive machine learning | Image segmentation and classification | Free, open source | Semi-automated; performance depends on user input |
| **AutoML platforms (**e.g., [Google AutoML](https://cloud.google.com/automl?hl=en), [H2O.ai](https://h2o.ai/)**)**  <https://cloud.google.com/automl?hl=en>  <https://h2o.ai/> | Predictive modeling in nephrology datasets | Automated ML pipelines | Development of predictive models without advanced coding | Mixed (free tiers / paid plans) | General-purpose; not pediatric nephrology-specific |

Note. This list is illustrative rather than exhaustive and subject to rapid evolution given the pace at which AI applications are evolving.
